# Exploring veterinarian and pet owner perspectives on the risk of antimicrobial resistance when feeding raw meat diets to dogs

**DOI:** 10.1002/vro2.70040

**Published:** 2026-07-09

**Authors:** Tamzin Furtado, Nicola Williams, Constance Gascoyne, Gina Pinchbeck, Genever Morgan

**Affiliations:** ^1^ Department of Livestock and One Health University of Liverpool Neston UK; ^2^ School of Veterinary Science University of Liverpool Neston UK

**Keywords:** antibiotic resistance, bacteria, dog, raw, raw meat diet

## Abstract

**Introduction:**

The feeding of raw meat diets (RMDs) to dogs has become increasingly popular in the UK in recent years. A major concern surrounding RMDs is the potential risks to humans and animals posed by the presence of potentially pathogenic bacteria, particularly for transmission of antimicrobial‐resistant (AMR) bacteria.

**Objectives:**

This study explores how veterinary professionals and owners conceptualise and communicate about the risk of AMR bacteria in RMDs.

**Methods:**

Focus groups and discussion forum data were used to explore veterinarian, nurse and dog owner views on the risk of AMR bacteria in RMDs. Six focus groups (three veterinary professionals and three dog owners) were conducted; additionally, 34 open‐access discussion forum threads around RMDs were collected. All data were analysed using a reflexive thematic analysis.

**Results:**

Results highlight that bacterial load (and therefore, potential for AMR) was perceived as a potential risk by participants from all groups, yet was generally considered less important than other concerns, such as nutrition. Veterinary professionals were concerned about AMR, and perceived issues, which could lead to the introduction of AMR bacteria at many stages of the production system. Raw‐feeding dog owners placed greater trust than veterinary professionals in the processes of diet creation and their dog's immune systems to manage those risks.

**Conclusions:**

This study will help dog health professionals and dog owners alike in reconciling communication between pro‐raw, neutral and cooked food feeders. Additionally, we identify that increased rigour in raw meat production systems could better protect consumers and public health.

## INTRODUCTION

Diets comprising primarily of raw meat ‘raw meat diets’ (RMDs) have gained popularity among pet owners in recent years, driven by beliefs that such diets offer multiple health benefits, such as improved coat quality, increased energy levels and better digestive health for their dogs.^1^, ^2^, ^3^ However, RMDs have been met with controversy and scepticism within the veterinary community due to a range of potential health risks,^4^ including the risk of bacterial contamination and related antimicrobial resistance (AMR).^4^, ^5^, ^6^, ^7^

A primary concern associated with RMDs is the presence of potentially harmful bacteria such as Salmonella spp., Listeria spp. and Escherichia coli, which can pose serious health risks.^7^, ^8^, ^9^ These pathogens cannot be eliminated through the preparation processes of raw diets,^10^, ^11^ and could lead to infections in both animals and humans.^6^, ^12^ Improper handling and storage of raw food can exacerbate these risks, creating potential public health issues in households where raw feeding is practiced without close attention to hygiene.^13^ These concerns are compounded by the risk of AMR, as exposure to contaminated raw diets may contribute to the spread of AMR bacteria and AMR determinants.^12^ In the context of RMD, there is concern that the contamination of products with AMR, particularly bacteria expressing multi‐drug resistance (resistance to at least one drug in three or more classes) could potentially lead to infections that are challenging to treat.^10^, ^11^

Raw meat diets can consist of pre‐prepared portions ready for feeding or can be prepared by owners at home by purchasing raw meat/components from pet stores, supermarkets, butchers, or abattoirs. In the UK, pre‐prepared RMDs are subject to regulation primarily by the Department for Environment, Food & Rural Affairs (DEFRA),^14^ who oversee animal feed safety, ensuring that raw pet food meets hygiene and safety standards under the Animal By‐Products Regulations, which control the processing and use of animal products in pet food.^13^ UK Pet Food (www.ukpetfood.org), a trade body, offers guidelines for producing safe and nutritionally balanced pet food, including raw diets, although membership and adherence are voluntary. Additionally, producers can choose to sign up to the RawSAFE (www.rawsafe.com), a group that provides audit and guidance specific to raw pet food, promoting best practices for handling, storage and hygiene. While these groups should provide some standardisation and safety, for example, via batch testing of bacteria in RMD, research has found concerning levels of AMR bacterial strains in raw food.^10^, ^11^, ^15^ Nevertheless, many pet owners continue to feed their dogs raw diets based on their perception of improved dog health, suggesting that consumers perceive diet‐related risk differently to the majority of the veterinary community and mistrust veterinary advice on the matter.^1^, ^2^

This study aims to explore the perspectives of both veterinary professionals (VPs) and pet owners on the risks associated with RMD, focusing on the issue of AMR and the dynamics of trust that underpin decision‐making processes. This will contribute to a deeper understanding of the factors that influence raw feeding practices and identify opportunities for improving communication and collaboration between stakeholders.

## METHODS

This study employed a qualitative research design to explore the perspectives of VPs and pet owners on the risk of bacteria and AMR associated with feeding RMDs to dogs. Focus groups were used to allow for in‐depth discussions and the gathering of rich, contextual data^16^, ^17^ from two groups of participants: dog owners who self‐described as feeding RMDs, and VPs who were practising in the UK. Participants were recruited via advertising online (for example, on social media platforms including open Facebook groups around dog ownership) and in local pet shops (both chains and independent stores), followed by snowball sampling. Participants applied to the study by filling in a brief demographic form including their experience with RMDs. The inclusion criteria for VPs included being a veterinarian or vet nurse working in the UK in small animal practice, with availability to take part in the focus group. The inclusion criteria for owners included being an English‐speaking, UK dog owner, over the age of 18 years, with availability to take part in the focus group. All applicants were subsequently invited to the interview.

Six focus groups were conducted during May‒July 2023: three with VPs (one in person at University of Liverpool and two online), and three with raw‐feeding dog owners (one in person at University of Liverpool and two online). In total, 21 VPs were involved, including 19 veterinarians and two nurses, representing a diverse range of experiences and veterinary practice settings (e.g., corporate, private, university‐owned and charity settings). The pet owner focus group comprised eight participants, representing a variety of backgrounds and feeding practices; participants owned one to four dogs, were all female, and held professional jobs (e.g., researcher and healthcare professional). Participants described feeding a mixture of commercial and home‐prepared raw diets, and had between 1 and 25 years’ experience of feeding raw.

For all groups, the focus groups were around 1.5 h in length; structure involved initial open discussion about participants’ experiences and beliefs about RMDs, followed by a 20‐min presentation about previous research conducted by several of the authors, around bacterial contamination of RMDs and prevalence of AMR‐*E. coli* (findings available^10^). Following questions about the findings, respondents were invited to ask questions and share their perspectives on the findings and how these might influence their views on raw feeding, if at all. The topic guide is available in the Supporting Information. The focus groups were led by T.F., an experienced facilitator and researcher employed at University of Liverpool, who is not a VP and who holds a neutral positionality regarding raw and cooked pet diets (owns pets currently fed non‐raw diets, but is open to other diets and has no biomedical or nutritional training), and has not been previously involved in studies about RMD. A co‐facilitator, G.M., was present in all focus groups to provide the presentation components and answer questions about them. G.M. is a small animal veterinarian who has conducted a PhD in RMDs for dogs. The positionality of the facilitators was briefly outlined at the beginning of the discussion. All focus groups were audio recorded to ensure accurate capture of the discussions.

Audio recordings from the focus groups were transcribed verbatim using a professional (human) transcription service. The transcripts were then reviewed for accuracy and anonymised. Brief field notes were captured during the focus groups, for example, to remind the facilitators of topics to return to at a suitable point; these were not used further in the analysis.

Alongside the focus groups, three open‐access public discussion forums^17^ around companion animals were identified as containing discussions surrounding the pros and cons of raw‐feeding. These online platforms are specific discussion forums for certain topics (e.g., pet owning); not social media platforms. Platform users post under avatars or pseudonyms and data are publicly available without needing to log in. For each forum platform, the terms ‘raw dog food’ were used to access the specific threads from January 2022 to July 2023. From the three different platforms, a total of 34 discussion threads were identified, including 809 comments overall. These were downloaded, further anonymised if any identifiable information such as place was mentioned, and analysed in the same manner as the focus group data.

Ethical approval for the study was obtained from the University of Liverpool Veterinary Ethics Committee (VREC 1326). Informed consent was obtained from all participants prior to their involvement in the focus groups. Discussion forum data were taken from anonymous open‐access discussion fora, with publicly available data, although single words have been changed in the quotes below to further protect poster's anonymity.

### Data analysis

A reflexive thematic analysis as outlined by Braun and Clarke^16^, ^18^, ^19^ was employed to analyse the data. Thematic analysis involves six interconnected phases of analysis in which the researcher systematically creates codes inductively from the data in order to identify, refine and consolidate themes. A symbolic interactionist perspective^20^, ^21^ was applied to understand how veterinarians and pet owners construct and interpret meanings around RMDs and AMR. T.F. coded focus group data, and C.G. coded forum data in separate files. Lumivero (NVivo 2020) was used to assist with the analysis. Additionally, visual ‘maps’ were created as part of the analytical process to explore how developing themes and codes interacted with one another, and how veterinarians and owners perceived the diet creation system. All coding was discussed within the team in group meetings at regular intervals to ensure a harmonised and consistent approach, and to discuss and resolve different application of codes. The findings from the three data types (focus groups from VPs, focus groups from owners and forum data) were then synthesised by exploring the alignment of key themes.

The CORE‐Q checklist was used to ensure that the methods and results have been presented fully (available in Supporting Information).

## RESULTS

Both VPs and owners felt a strong sense of responsibility for maintaining the optimum health of the dog, although their potential mechanism for expressing this responsibility differed: owners through a desire to make good choices to maximise the dog's wellbeing, and VPs, by communicating with the owner about choices that they perceived would maximise dog health. Differences in perspective arose predominantly due to the trust placed on different areas of the systems of RMD formulation, production and feeding leading to diverging views and culminating in difficulties communicating over these diets.

### Veterinary professional's perspective on RMD system

Veterinary professionals who took part in this project had mixed views on the feeding of RMDs, predominantly being cautious of RMDs but not considering themselves ‘anti’‐raw:

I'm ambivalent about raw food [veterinarian, focus group 3]

There's such little evidence there to say one is better than the other. I don't think you can make those judgements saying, ‘This food is better for this dog’. [veterinary nurse, focus group 3]

Via their training, VPs had an understanding of the system of dog food creation, having worked with production animals on‐farm, witnessed slaughter and meat processing, and being at least partly familiar with guidelines such as those produced by DEFRA and UK Pet Food. However, while their knowledge of meat production and dog health was high, their confidence around dog nutrition was variable; several had undergone specific training, but those who had not felt that they lacked adequate knowledge:

I would definitely usually just go, ‘Right, what you need is what we've got on the shelf here, because someone else has thought about it’, and I can go to the book and I can say, ‘That looks about right. There you go’. [veterinarian, focus group 2]

Veterinary professionals’ main concern about RMDs tended to be the nutritional profile of meals provided, with many being concerned that RMDs had a higher chance of being ‘unbalanced’; that is, lacking the full requirement of nutrients required by dogs. However, VPs were also concerned by other perceived issues such as physical harm to the dog from components of the diet (e.g., the dog ingesting bones), as well as the bacterial risk posed by RMDs. Many participants could cite examples of dogs seen at their practice who had clinical problems arising as a result of raw feeding, although they also recognised that their views might be biased by the animals who visited their clinics with medical issues.

The presence of excessive or harmful bacteria in RMDs was a concern for VPs at both a local level (presenting risk for the dog owner and their family) and as a wider One Health concern. The invisibility and pervasive nature of bacteria meant that VPs perceived the presence of harmful bacteria as an inevitable yet hidden risk. Thus, VP's advice was to avoid the presence of excessive bacteria wherever possible to protect dogs and owners from the risk of clinical disease. They perceived that this advice had the additional benefit of reducing the use of antimicrobials:

… about our code of conduct, if you're a vet or a nurse, and that one health aspect—I think the one health part really, really, worries me with antimicrobial resistance. I think we've got a huge role, as VPs, to ensure that we preserve the antimicrobials that we have. We need to look at the influence that raw feeding does have on this [veterinary nurse, focus group 3]

Veterinary professionals described concerns about excessive bacteria within RMDs due to a lack of trust in the way in which bacterial risk was mitigated throughout the system of diet creation and feeding, from meat processing at slaughter through to management of the dogs’ faeces in the home. While cooking meat would help to mitigate these risks, VPs perceived that the current system had the potential for creating diets that contained excessive contaminants, which were then misrepresented to owners, who themselves might have poor awareness of the risks. For example, several VPs discussed the potential for issues across the farming, production and distribution system that would lead to increased risk:

Antimicrobial resistance that is present in that meat from faecal contamination is already present in the farmed animal [veterinarian, nutritionist, focus group 2]

[As part of University] we had to go and do two weeks in a slaughterhouse … I, immediately after that, advised against all my relatives ever eating rare steaks ever again after I saw carcasses touching each other and all the kind of … What the meat inspectors had to put up with in terms of trying to get the companies to do the right thing …—That's why I've never, actually, directly recommended anybody put their pet on raw food. That's what actually holds me back from it, my own experience in the slaughterhouse and seeing what happens to the food that goes into the human food chain [veterinarian, focus group 1]

Veterinary professionals distrusted many of the companies that produce RMDs, perceiving that these companies might aim to meet consumer demand without adequate regard for bacterial safety. Regulatory bodies were perceived as having less authority and oversight over the marketing of RMDs compared to cooked food, and the recent explosion in new companies selling raw food resulted in problems tracking and managing company behaviour. Veterinary professionals described ‘scepticism’ towards marketing claims and companies that promoted products in ways that they felt were misleading to consumers, with the aim of financial gain rather than dog health:

You don't want to judge people who want to do the best but I do feel very judgy towards some of those companies who I don't think want to do the best, from my point of view anyway [veterinarian, focus group 3]

Once products had been purchased by owners, VPs also perceived there were potential hazards within the later stages of the RMD system. Veterinary professionals perceived that raw‐feeding owners may not be aware of the risks of handling, storing and managing the environment of RMDs and raw‐fed dogs. Their concerns were predominantly around owners’ lack of knowledge and concern around bacteria and hygiene management, which were often related to the invisibility of bacteria and minor bacterial illnesses:

Well, that's the one that's going to come up in response as, ‘Yeah, but the owners won't get sick’. It's like, ‘yeah, but … when they do get sick, they'll die’ [veterinarian, focus group 2]

Notably, VPs perceived that dogs fed RMD could themselves act as a bacterial vector and thus a risk for contamination of others in the home; for example, because of bacteria in saliva, which might be transferred to their coats or paws during self‐grooming, or to other objects such as dog toys. As a result, VPs often considered the environment of dogs fed RMDs to be potentially hazardous and harmful; as one veterinarian described ‘like keeping my raw chicken chopping board around the house [veterinarian, focus group 2]’.

The lack of trust in the system of RMD diet creation was compounded by VP's high trust in scientific research about the extent of bacterial AMR. In weighing up the risks of these factors compared with the potential benefits of feeding raw, VPs in this project perceived cooked diets to be lower risk:

If there is no clear benefit, why are we taking the risks? If there were clear benefits, it might be a trade‐off. I haven't found them yet, apart from that one study that said if you feed Westies a little bit of raw food before they are, I think, 16 weeks old they might express atopy a little bit less frequently [veterinarian, focus group 1]

It was notable that VPs did not blindly trust cooked food producers either, but perceived that the bacterial risk was substantially reduced through the process of cooking. Overall, VPs were frequently frustrated by the polarisation around feeding practices in dogs:

The thing that bothers me the most is that we have those polarised views and neither of them are correct. And we get people saying you should always feed raw and people saying you should never feed raw, and the reasons given on both ends tend to be pretty poor [veterinarian, focus group 2]

### Owners’ perspective on RMD system

Owners who participated in the focus group study were generally knowledgeable about the RMD production systems, and were invested in considering issues such as production animal management (e.g., antibiotic use in farm animals), handling of raw meat and product formulation. RMD‐feeding owners were generally aware of the risk of excessive or harmful bacteria on meat, but held diverse views about how this should be managed, and the potential for harm. While some described fastidious processes of managing hygiene in the house, others perceived that eliminating harmful bacteria (as suggested by the veterinary approach) could be unhealthy in itself; one owner described exposing bodies to ‘clean dirt’ [dog owner, focus group 1], referring to some level of bacterial exposure, and another stated:

We need bacteria and there are good and bad ones. So I assume that if you're actually killing bad ones (for example by cooking the diet or hygiene practices), you're also killing good ones on that. [dog owner, focus group 2]

This owner described that the benefits of the ‘good’ bacteria outweighed the risks of the ‘bad’ bacteria, leaving them confident in their decision to use RMD. Additionally, many RMD‐feeding owners described trusting processes such as freezing to reduce bacteria, or described a perception that the canine body itself is well‐equipped to manage the risks of excessive or harmful bacteria:

Carnivores have high levels of stomach acidity which helps them to kill harmful bacteria from their prey [discussion forum poster; board 1, thread 4, comment 3]

Raw is fine for dogs, their intestines are a lot shorter than humans so food passes quicker; therefore, bacteria doesn't form so is safe [discussion forum poster; board 1, thread 4, comment 18]

The dog's own behaviour and choices were often perceived by owners to constitute evidence of dogs’ ability to manage bacteria, because they frequently chose to eat things that were ‘disgusting’ [dog owner, focus group 2] and potentially contaminated, yet rarely became ill:

My girl fished a bloated dead rat out of a river. I don't know how long it had been there, but I've never smelt anything so horrific. I could still smell it on her. I got it off her. She didn't consume it, but she had a good chew [dog owner, focus group 1]

I know some mention the bacterial risks of raw meat, and they are real, but you've got to balance that risk with the fact that some dogs will eat bird and dog poop and come out happy as a clam [dog owner, focus group 3]

Many RMD‐feeding owners therefore placed trust in the dogs’ ability to make species‐appropriate choices, and the dog's body as a mechanism that could reduce risk. They located areas of risk of bacterial contamination as kitchen areas where food was defrosted and prepared, dog food bowls, and—outside of the kitchen—dog faeces. Fewer owners were concerned about risk of bacterial contamination from the dog's saliva, or its presence on dog toys/the dog's coat after self‐grooming.

Unlike VPs, RMD‐feeding owners often placed trust within the RMD‐producing companies in preference to the producers of cooked diets, who were perceived as having commercial gain at the heart of their practice and thereby being morally questionable:

We have these three companies who have the most abhorrent rap sheet, are notorious polluters and use child slavery, yet we think they have our pet's health as their top priority. I don't buy their products neither for myself, nor will I buy them for my puppy [discussion forum poster; board 3, post 11, comment 5]

The commercial power of these organisations was considered to be such that they might affect other areas of the system, for example, by influencing veterinarians in practice, or research performed. As a result, owners often placed little trust in veterinary‐led guidelines and research:

To be fair, the World Small Animal Veterinary Association (WSAVA) is primarily sponsored by The Big Three [companies], and they're notoriously gatekeeping the dog food industry by spending billions on everything from vet schools to scientific studies [discussion forum poster; board 2, thread 1, comment 4]

Research is often done for things like the kibble companies and stuff like that, often done in‐house or funded by them because they've got the money to do that [dog owner; focus group 3]

Along with this mistrust and scepticism of the commercial components of the non‐raw system, owners lacked trust in VP's knowledge of nutrition because of a perceived lack of nutritional training within the veterinary curricula.

Other stakeholders working in raw feeding (including influencers and manufacturers) were often perceived as being more transparent, and therefore more trustworthy, than stakeholders working in the cooked diet industry. This was partly due to the visibility of diet formulation, as well as the fact that influencers encouraged owners to assess evidence themselves:

They say, ‘Do your research. Look for the proper evidence’ [dog owner, focus group 1]

Pet shops that promoted RMDs were also thought to give supported, tailored advice and were often perceived as being knowledgeable about nutrition, providing both practical and social support. Their allyship could result in a trusted relationship, giving confidence to dog owners in their practice and approach.

Additionally, owners often perceived the actions of their dog feeding practices to have only local relevance, without wider implications for people outside their household:

My dogs are fed raw and it doesn't affect the public in any way [discussion forum poster, board 1, thread 4, comment 2]

If you're challenging the welfare of the dog versus humanity, there are going to be a lot of strong emotions. I like my dogs more than all the people I know. I love my dogs more than the moon itself. So I would never change my opinion of feeding biologically appropriate food [dog owner, focus group 2]

However, this was not a unanimous view, and several owners were cognizant of public health risk. For example, one raw‐feeding owner had worked in pet food production, and described:

I think the raw feeding community as a whole are trying to use that to justify not thinking about the future of the resistant bacteria. And I don't think that those people should be burying their heads in the sand [dog owner, focus group 2]

### Veterinary consult: a potential clash of world views

Raw meat diet feeders in the forums and discussion groups alike reported mixed conversations with veterinarians, with some VPs supporting decisions to feed raw and others recommending cooked diets; similarly, veterinarians reported mixed interactions with owners around raw feeding. Nevertheless, the veterinary consult was expressed by both parties as a potentially challenging encounter, with both owners and VPs being aware prior to the consult that the other may have strong views contradictory to their own. Both groups described feeling responsible for the health of the dog and therefore entering discussions about raw feeding with pre‐conceived ideas about the other party, perceptions about how the other was likely to behave and strategies for managing the interaction.

Raw‐feeding owners who disagreed with veterinarians were perceived by VPs as challenging the expected norms and power dynamics of a veterinary consult, in which the veterinarian is typically cast as an expert whose advice should be followed. RMD‐feeding owners, by contrast, often viewed their feeding‐related knowledge as superior to the veterinarians, viewed themselves as primary decision makers (‘it's my dog’ [dog owner, focus group 1]) and often distrusted diet‐related advice they received from veterinarians. Language used to describe the veterinary approach to communication about raw sometimes referenced VPs being patronising, paternalistic and dismissive:

It can be quite patronising, can't it? Like, about the bacteria and things like that. ‘It's got bacteria in’, and it's, like, ‘Yes, I understand that. I understand that raw meat has bacteria, and I understand that I need to take precautions’, but I think they can act like you don't know that [dog owner, focus group 1]

[describing the veterinary approach] Look what you're doing. Look how awful an owner you are for making this decision. Which it can definitely feel like, whether or not it's being put out in that way [dog owner, focus group 2]

This approach could be problematic because many of the RMD‐feeding owners described themselves as invested in learning, discussing evidence‐based practice, and making informed choices about feeding. A directive approach could be perceived as disrespectful of their knowledge. For example, one participant described asking a VP on social media for more information about nutritional guidelines that the VP had mentioned, and being rejected due to that person's perception that they would not be able to interpret the evidence:

The attitude was: ‘but you wouldn't understand’. [But] there are quite a lot of intelligent, educated people out there that aren't vets [dog owner, focus group 1]

RMD‐feeding owners very frequently mentioned seeking research papers and evidence, and referenced paywalls and lack of open‐access science as a barrier to them increasing their knowledge, despite their passion for learning:

Because National Institute for Health and Care Excellence (NICE) guidelines for human healthcare are widely accessible, but you try getting hold of a veterinary guideline, it is incredibly difficult … okay, there are some people that won't understand NICE guidelines, but a lot of us highly motivated dog owners would love to be able to access the guidelines, and it might even actually help us inform on shared decision‐making with our vets [dog owner, focus group 2]

It was suggested that this potentially problematic communication dynamic can be a specific convention of veterinarian‒client consultations, with nurses perceived as having a more conversant communication style, resulting in them sometimes being more trusted by clients; therefore, they were seen as potentially more successful at discussing pet diets with owners. However, veterinarians themselves frequently needed to cover these topics in routine consults. In order to manage the communication within this challenged power dynamic without conflict, both groups used different strategies. For example, some owners did not tell the truth to veterinarians in order to avoid potential judgement:

People would say to me, ‘I told my vet that I used the antibiotics, but all I did was change to a raw diet. But I'm too scared to tell my vet the real truth’ [Discussion forum poster; board 3, discussion 3, comment 6]

However, the VPs who took part in the focus group discussions were well aware of the potential difficulties of communication about this issue, and that owners might not ‘admit’ to controversial pet care choices. Veterinary professionals reported feeling wary of discussing RMDs for risk of alienating clients, breaking trust, and further reducing communication:

‘I've got general practice clients who just really feed terrible raw food, really badly balanced, really terrible raw food, but if I continue to hassle them all I'll do is alienate them and they won't come in at all’ [veterinarian, focus group 2]

Ultimately, these discussions led to negotiation of responsibility around dog diets, with the role of VPs challenged in comparison with their role in relation to many other medical or health issues. Veterinary professionals reported that this issue led them to reconsider the power they held within each consult, sometimes relinquishing their responsibility:

My fear really when I'm speaking to those people is that they'll disappear … I feel the same about fat dogs. You should give good advice to the extent that you can, but there's got to be a line whereby you say, ‘okay, they've made that decision and I just have to suck it up’ [veterinarian, focus group 2]

### Does anything need to change?

The presentation given as part of the focus groups reported recent research in which high levels of AMR bacteria were identified in commercial pre‐prepared RMD products. Participants in all focus groups were concerned about the high levels of AMR bacteria found in the study:

One of the things that really hit home for me, we already know that it is a risk and we thought it was being tested before and regulated, to an extent. And clearly, these results show something quite different to what we think is happening at the moment [veterinarian, focus group 1]

Your research raises an awareness of a level of concern that it was at the back of the head, you know it's a thing, but maybe not at the forefront [dog owner, focus group 2]

When you see antimicrobial resistance to more than three antibiotics, then you think ah … I think that's just that really naïve thing of you think, ‘Well, there's bugs everywhere’, but actually to think, like you say, of somebody putting it in their fridge with antimicrobial resistance to more than three antibiotics, then it's a whole different ball game, isn't it? It's like actually that's massive [veterinarian and nutritionist, focus group 2]

As a result, both RMD‐feeding owners and VPs discussed the need for change in how bacterial risk should be communicated to dog owners. While no RMD‐feeding owners described wanting to change their dog food preferences, there was extensive discussion in each group about mitigating risk in other ways.

Both VPs and owners highlighted that consumers were frequently problematised around raw feeding and hygiene management, yet the production system was failing them by supplying a product with poor safety standards. Participants were unequivocal about the need for system‐level and policy‐level change in relation to this issue in order to protect consumers:

I think largely it needs to be focused at, if you're going to produce raw, here are the risks that we've discovered on the production of raw, what can you do now to remove those for your buyers? Because that's the responsibility you have as a profiting‐company [dog owner, focus group 2]

There should be more onus on the entire food chain and the whole farming industry rather than just on the end consumer. Like, how does that antimicrobial‐resistant bacteria get there? [dog owner, focus group 1]

I probably view the companies as bad people, in this situation. They're not doing it properly [veterinarian, focus group 1]

As such, the lack of transparency over food production practices and reporting standards which allowed potentially harmful practice were seen as areas where potential change could be effective.

Study contributors also described the need for respectful communication, which empowered owners, allowed for their independent decision making, and provided pragmatic support:

If I go to a vet and I say my dog is raw‐fed and the reaction is, ‘Did you see that study from Liverpool about bacteria?’ immediately, immediately I'm switched off … Rather than going, ‘Have you considered X, Y, Z. Here is how you can do it safely and appropriately, if that is what you want to do’ [dog owner, focus group 2]

Additionally, providing respectful but evidence‐based information open access was important, with guidelines and study results being available for consumers:

If … instead of just consulting Doctor Google or Vet Google, you could go to the British Small Animal Veterinary Association (BSAVA) website, see what the guideline says, and formulate your questions and then have an informed discussion with your vet [dog owner, focus group 1]

Veterinary professionals described using a range of approaches to discuss raw feeding with clients and to communicate about risk while respecting the owners’ autonomy. These included presenting themselves as open‐minded about diet choices, making sure that owner autonomy was respected, contextualising risk with real‐life examples or human analogies such as ‘hospital superbugs’, giving owners resources to read in their own time, and discussing ways owners could minimise risk.

## DISCUSSION

This study has explored raw feeding as an issue about which VPs and clients frequently have divergent opinions. While all stakeholders wanted to maximise dog health, their perception of risk around dog health, human health and public health was often in direct contrast.

Focus group participants unanimously acknowledged that there are problems with the way that the RMD system operates, with potentially unsafe products being marketed to consumers. Numerous studies have found harmful levels of bacteria in raw food, poor information on packaging, poor nutritional balance and misleading language.^5^, ^7^, ^10^, ^11^, ^15^, ^22^ So far, efforts at reducing risk have been aimed predominantly at warning consumers, leading to communication challenges in practice. However, this approach overlooks the wider problem of products being marketed with unacceptable levels of contaminants present, despite existing guidelines, testing and reporting. This warrants urgent attention from policy makers, since redesigned systems could have extensive benefits for minimising AMR. These systems might include components that minimise contamination and have more stringent regulation, traceability, testing, reporting, labelling and consequences for companies whose products are contaminated beyond appropriate levels.^22^, ^23^, ^24^

While raw‐feeding owners represented a wide range of views, a frequent commonality was their wish to source and evaluate evidence themselves.^13^, ^25^, ^26^ The lack of access to research findings, guidelines and veterinary literature was therefore described as constituting a major obstacle in maintaining trust between those working in veterinary medicine and research, and the dog owning public. Other studies have suggested that online advice‐seeking is important to owners; thus, providing reliable, accessible and trustworthy information would facilitate veterinary communication.^25^, ^27^ We suggest that it is imperative that veterinary research and guidelines are made open access and are complemented with lay‐friendly knowledge summaries. Additionally, those working in veterinary medicine or research could share information about how keen dog owners can themselves find, read and evaluate clinical studies, including assessing bias.

In this study, both VPs and owners sometimes expected discussions about raw feeding to be combative, since each group held pre‐conceived expectations about the other. However, we found a very high level of in‐group diversity; participants’ beliefs varied widely, with differing perspectives around the pros and cons of raw feeding, bacterial risk and hygiene management. In other fields, researchers have studied improved communication between groups who believe that they have opposing values, noting that finding values which accord between groups, and reframing messages with values that are important to the other group, are key to more pragmatic discussions.^28^ Therefore, it is hoped that information from this study will help both groups to find common ground and reduce polarisation.

Our study identified that communication about raw feeding sometimes led to a reconfiguration of power dynamics between VPs and raw‐feeding owners. Other issues have also been identified as leading to complex clashes of value between VPs and clients who behave in ways that do not fit within a veterinary paradigm. For example, owners who choose breeds of animal known to have health issues,^29^ or who do not put their animals on a diet despite being told they are overweight.^30^ These issues reveal a complex tension at the heart of veterinary medicine. Veterinarians swear an oath ‘to ensure the health and welfare of animals committed to my care’,^31^ and one study suggested that ‘liability issues could arise if veterinarians do not discuss potential risks’.^22^ Nevertheless, there are times when VPs perceive the potential for harm, but cannot intervene in the animal's health beyond communicating with owners.^32^ Working within this paradox is complex and veterinarians are reported to feel high levels of frustration around the emotional burden of balancing apparently competing priorities, while aiming not to alienate clients.^32^, ^33^, ^34^

Discussions about raw feeding can be further complicated by the fact that studies have shown that veterinary communication is frequently paternalistic and directive, despite this being a relatively unsuccessful communicative approach.^35^, ^36^, ^37^ In the present study, the VPs who took part gave multiple reports of ways that they communicated about RMDs in a collaborative, supportive manner, in line with the approaches owners described wanting their veterinarians to adopt. These approaches are aligned with good practices around communications which aim to empower owners and collaborate in shared decision making.^35^, ^36^ Interestingly, veterinary nurses were also highlighted in this study as ideal information sharers because they are perceived to have more conversant communication and approachability. Thus, we suggest that communicative approaches that empower owners as decision makers are ideal and that veterinary nurses who are interested in nutrition are given opportunities to support or lead discussions about RMDs.

### Limitations

This study is limited by the potential sampling and response bias from participants, who may have taken part because they held particular views. For example, the best‐practice communication approaches described by our participant VPs differ from the evidence in the literature around the frequent use of directive veterinary communication.^35^, ^36^, ^37^ Indeed, several of the VPs who took part had a particular interest in learning more about raw feeding. Nevertheless, participants in all focus groups represented a range of diverse views, practices and behaviours, and thus we consider that the results will be of use to a range of stakeholders with an interest in this topic. A second limitation of the methodology is that we rely on self‐reported behaviours, including feeding/hygiene practices, and also communication approaches. Observational approaches could provide additional data around how people behave in real life, and further insight into areas of complexity and potential behavioural intervention to facilitate interactions between clients and veterinarians, as well as safety.

## CONCLUSION

This study is the first, to our knowledge, to explore and compare veterinarian and client perceptions of RMD using qualitative methodologies. We explored divergent views around raw feeding using a system‐based approach, which has allowed us to identify the trust and mistrust stakeholders hold around aspects of the system of RMD creation and feeding. Indeed, we have identified that aspects of the system are particularly problematic, since the diets being marketed have been described as unsafe, with consumers rather than producers getting the blame. We consider that it is an urgent requirement for there to be policy change to better inform and protect consumers. This should promote improved safety standards in the production and processing and supply chain for RMDs, better traceability of ingredients, improved packaging and clearer labelling.

We have identified that discussing RMDs can highlight veterinarians’ lack of control around owner behaviour, as well as owners’ desire to learn and make informed decisions around dog health themselves. As such, we consider that it is important that the veterinary industry prioritises transparency, open‐access publication, lay‐summaries of information and guidelines, and information about how clients can assess research evidence themselves. This could also benefit VPs, by facilitating relationships with clients and communication during consults.

Additionally, VPs and clients alike should be mindful of the vast diversity of knowledge and approaches held by individuals, and be careful of imposing preconceived ideas about other groups. Communicative approaches that emphasise respect, autonomy and empowerment should be embraced.

## AUTHOR CONTRIBUTIONS

*Conceptualisation*: Nicola Williams, Genever Morgan and Gina Pinchbeck. *Methodology*: Tamzin Furtado, Nicola Williams, Constance Gascoyne, Gina Pinchbeck and Genever Morgan. *Data analysis*: Tamzin Furtado and Constance Gascoyne. *Main manuscript writing*: Tamzin Furtado. *Editing*: Tamzin Furtado, Nicola Williams, Constance Gascoyne, Gina Pinchbeck and Genever Morgan. *Acquiring funding*: Nicola Williams and Genever Morgan.

## CONFLICTS OF INTEREST

This study was funded by a University of Liverpool Policy Support Fund, which is in turn funded by Research England. The authors declare they have no conflicts of interest.

## ETHICS STATEMENT

Ethical approval for the study was obtained from the University of Liverpool Veterinary Ethics Committee (VREC 1326). Informed consent was obtained from all participants prior to their involvement in the focus groups. Participants were assured of the confidentiality and anonymity of their responses, and right to withdraw from the study. Discussion forum data were taken from anonymous open‐access discussion fora, with publicly available data, although single words have been changed in the quotes below to further protect each poster's anonymity.

## Supporting information



Supporting Information

Supporting Information

Supporting Information

## Data Availability

Data will be made available to researchers with appropriate ethics approval upon request.
