# Supplementary material for: Exploring veterinarian and pet owner perspectives on the risk of antimicrobial resistance when feeding raw meat diets to dogs
Source: Vet Rec Open. 2026 Jul 9;13(2):e70040. doi: 10.1002/vro2.70040 (PMC13347624; doi:10.1002/vro2.70040)
Supplement: Supplementary file 1 — Supporting Information [file VRO2-13-e70040-s001.docx]

**Supplementary item 1: Focus Group Questions**

Focus group 1: Dog owners

*Time block 1 –* half an hour

Question 1: to start with, we’d like to go around the room and hear a little bit about you, your dog and what prompted you to choose a raw diet for your dog(s)?

[followed by some discussion: a lot of the below may be covered in their initial intros, so kept here as a reminder – if covered, go straight to Q2. If not, include these in discussion]

Prompt 1: What do you think are the main benefits of feeding raw to your dog?

Prompt 2: Do you have any concerns surrounding feeding a raw diet to your dog?

Prompt 3: Where did you go for information regarding your dog’s diet choice?/ Could you tell us who (or what) was most influential to you when thinking about what to feed your dog?

[optional] Question 3: What are you hoping to learn from the session today/this evening (if anything)?

**Coffee/presentation** [Show presentation – 15/20 mins]

*Time block 2*

Question 1: What do you think of the findings from the study? [15/20 mins]

Prompt 1: Is there anything surprising to you in the results?

Prompt 2: Do the results of the study make you feel differently about raw feeding at all?

Question 3: After this discussion, is there anything you would do anything differently in relation to where you buy your food, how you store it, or how you clean up after your dog? [15 mins]

Question 4: What areas for future research around dog diets in general would you like to see? [10 mins]

Prompt: In particular, what areas for research around feeding a raw diet would you like to see?

Focus Group 2: Veterinary professionals

*Time block 1*

Question 1: To get us started, can you tell us a bit about you, your role, and your interest in raw feeding? [15 mins]

Prompt 1: What do you think are the main risks?

Prompt 2: Do you think there are any benefits?

Prompt 3: Do you think there are any public health concerns regarding raw diets?

Question 2: Can you tell us about your experiences with regards to communication with clients about raw diets? [15 min]

Prompt 1: What do you think clients believe (understand?) about raw diets?

Prompt 1: Do you encounter any difficulties with communication surrounding raw diets?

Prompt 2: Do you think clients are aware of the public health risks?

Question 3: What are you hoping to learn from the session today/this evening (if anything)?

**Coffee/presentation**

*Time block 2*

Question 1: What do you think of the findings from the study?

Prompt 1: Is there anything surprising to you in the results?

Prompt 2: What do you think of the strength of the evidence?
