# Supplementary material for: Exploring veterinarian and pet owner perspectives on the risk of antimicrobial resistance when feeding raw meat diets to dogs
Source: Vet Rec Open. 2026 Jul 9;13(2):e70040. doi: 10.1002/vro2.70040 (PMC13347624; doi:10.1002/vro2.70040)
Supplement: Supplementary file 2 — Supporting Information [file VRO2-13-e70040-s003.docx]

| **Overall themes** | **Codes** |
| --- | --- |
| Negotiating trust and risk in feeding decisions | Perceptions of the risk presented by bacteria |
|  | Navigating clinical evidence (and lack thereof) |
|  | A dynamic journey of decisions |
|  | Sources of advice |
|  | Weighing up risk |
| Imagining food pathways as part of food choice | The impact of farming processes |
|  | The impact of meat processing |
|  | The impact of manufacturers choices |
|  | The impact of my choices in the home |
|  | Comparing the risk presented by human and dog diets |
| Making the right choice for my dog | Dog agency and preference |
|  | Knowing my dog as an individual |
|  | Outcomes of my choice |
|  | The supportive community around RMDs |
|  | Personal perceptions of a “good diet” |

Supplementary item 2: Coding table of the themes and codes arising from data involving dog owners (focus groups and forum data)
