# Supplementary material for: Exploring veterinarian and pet owner perspectives on the risk of antimicrobial resistance when feeding raw meat diets to dogs
Source: Vet Rec Open. 2026 Jul 9;13(2):e70040. doi: 10.1002/vro2.70040 (PMC13347624; doi:10.1002/vro2.70040)
Supplement: Supplementary file 3 — Supporting Information [file VRO2-13-e70040-s002.docx]

| **Overall theme** | **Sub-codes** |
| --- | --- |
| Professional responsibilities as a vet | Critical evaluation of information |
|  | Managing bacteria as a pervasive omnipresence |
|  | Making the right choice for the dog |
|  | Managing multispecies safety |
|  | Managing uncertainty |
| Imagining food systems and their impact | The impact of farming processes |
|  | The impact of meat processing |
|  | The impact of manufacturers choices |
|  | The impact of owner choices in the home |
|  | The dog as a conduit for harmful bacteria |
| Navigating opposing belief systems | The consult as a cite for the clash of world views |
|  | Managing mistrust in vets |
|  | Pro-raw popularisation and social pressure on owners |
|  | Communication approaches for discussing risk |

Supplementary item 3: Coding table of the themes and codes arising from data involving veterinary professionals (focus group data)
